# A Systematic Review of Collective Evidences Investigating the Effect of Diabetes Monitoring Systems and Their Application in Health Care

**DOI:** 10.3389/fendo.2021.636959

**Published:** 2021-03-16

**Authors:** Maria Kamusheva, Konstantin Tachkov, Maria Dimitrova, Zornitsa Mitkova, Gema García-Sáez, M. Elena Hernando, Wim Goettsch, Guenka Petrova

**Affiliations:** ^1^Faculty of Pharmacy, Medical University of Sofia, Sofia, Bulgaria; ^2^Bioengineering and Telemedicine Group, Centro de Tecnología Biomédica, Escuela Técnica Superior de Ingenieros de Telecomunicación, Universidad Politécnica de Madrid, Madrid, Spain; ^3^CIBER-BBN: Networking Research Centre for Bioengineering, Biomaterials and Nanomedicine, Madrid, Spain; ^4^Utrecht Centre for Pharmaceutical Policy, Division of Pharmacoepidemiology and Clinical Pharmacology, Utrecht Institute for Pharmaceutical Sciences (UIPS), Utrecht University, Utrecht, Netherlands; ^5^National Health Care Institute (ZIN), Diemen, Netherlands

**Keywords:** diabetes monitoring systems, diabetes, glucose control, systematic review, personalized approach

## Abstract

**Introduction:**

Diabetes monitoring systems (DMS) are a possible approach for regular control of glucose levels in patients with Type 1 or 2 diabetes in order to improve therapeutic outcomes or to identify and modify inappropriate patient behaviors in a timely manner. Despite the significant number of studies observing the DMS, no collective evidence is available about the effect of all devices.

**Goal:**

To review and consolidate evidences from multiple systematic reviews on the diabetes monitoring systems and the outcomes achieved.

**Materials and methods:**

Internet-based search in PubMed, EMBASE, and Cochrane was performed to identify all studies relevant to the research question. The data regarding type of intervention, type of diabetes mellitus, type of study, change in clinical parameter(s), or another relevant outcome were extracted and summarized.

**Results:**

Thirty-three out of 1,495 initially identified studies, involving more than 44,100 patients with Type 1, Type 2, or gestational diabetes for real-time or retrospective Continuous Glucose Monitoring (CGMS), Sensor Augmented Pump Therapy (SAPT), Self-monitoring Blood Glucose (SMBG), Continuous subcutaneous insulin infusion (CSII), Flash Glucose Monitoring (FGM), Closed-loop systems and telemonitoring, were included. Most of the studies observed small nominal effectiveness of DMS. In total 11 systematic reviews and 15 meta-analyses, with most focusing on patients with Type 1 diabetes (10 and 6, respectively), reported a reduction in glycated hemoglobin (HbA1c) levels from 0.17 to 0.70% after use of DMS.

**Conclusion:**

Current systematic review of already published systematic reviews and meta-analyses suggests that no statistically significant difference exists between the values of HbA1c as a result of application of any type of DMS. The changes in HbA1c values, number and frequency of hypoglycemic episodes, and time in glucose range are the most valuable for assessing the appropriateness and effectiveness of DMS. Future more comprehensive studies assessing the effectiveness, cost-effectiveness, and comparative effectiveness of DMS are needed to stratify them for the most suitable diabetes patients’ subgroups.

## Introduction

Diabetes mellitus is a lifelong, chronic metabolic disease leading to various complications. It affects significant number of people worldwide as the newly diagnosed cases are increasing rapidly. That makes diabetes a global epidemic and a major cause of morbidity and mortality (1, 2). Being difficult to treat and expensive to manage, diabetes could be defined as a demanding and fast-growing problem for healthcare systems (3).

Several challenges exist for ensuring a better control of patients with diabetes mellitus. First of all, the most appropriate pharmacological treatment for every patient should be ensured based on patient’s personal characteristics and needs. A high level of adherence to therapy should be provided and periodically reassessed in order to achieve the treatment goals: adequate glycemic control with low risk of complications. Therefore, continuous and strict monitoring of patients’ condition, focusing on regular assessment of glycemic control, as HbA1c levels and blood glucose levels, should be performed and the most appropriate personalized, cost-effective method for continuous monitoring should be selected. As a result, an adequate and optimal resources allocation could be provided for every health care system.

**Figure 1:**
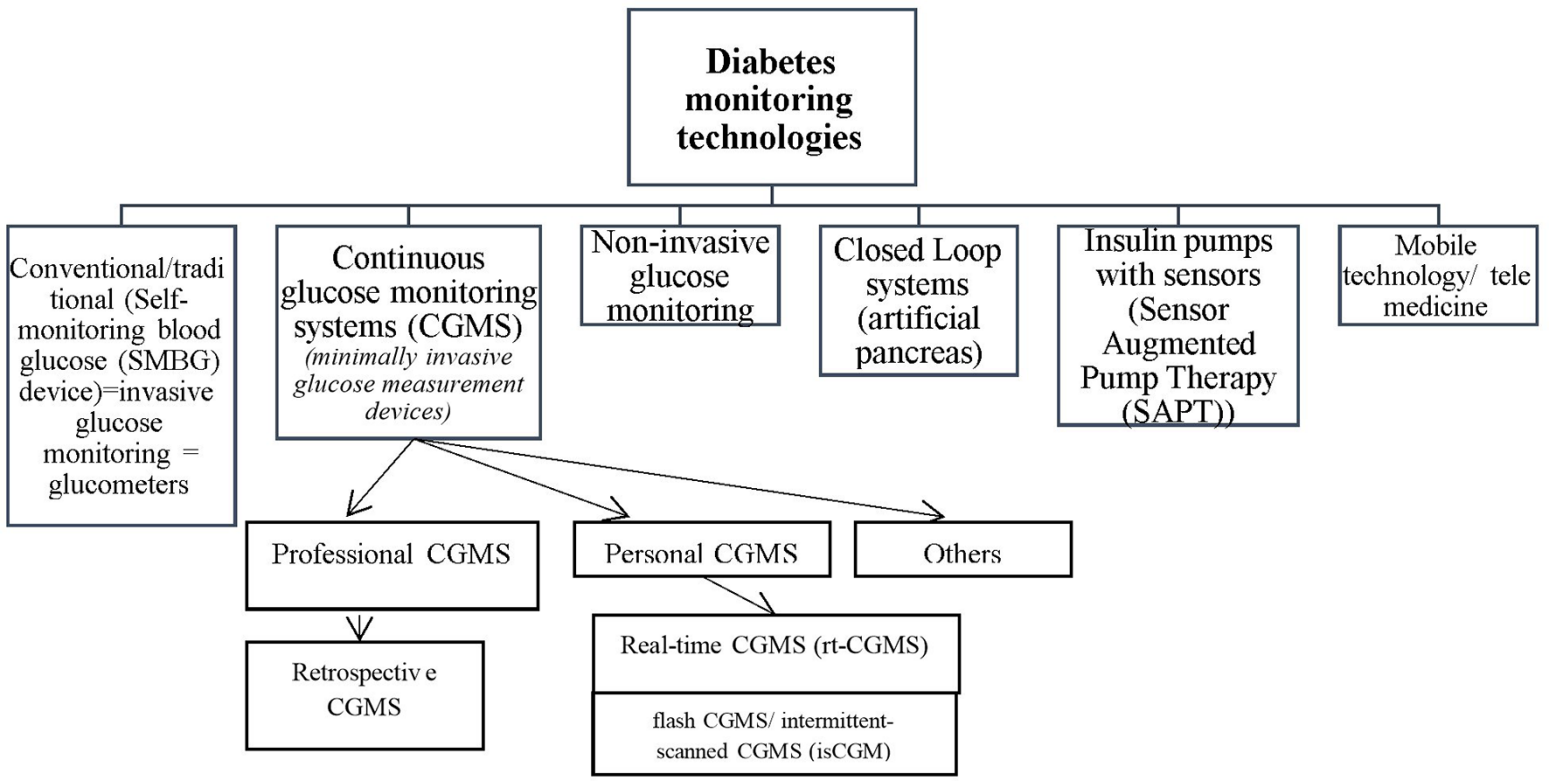
Classification of diabetes monitoring technologies.

Consistent engagement of patients within the process of effective management and control of glucose levels correlates with optimal health outcomes. Healthy eating, physical activity programs, adherence, and close monitoring are some of the most important self-management actions for the purpose of successful treatment (4). Glucose monitoring systems (GMS) are devices which provide information about glucose values ensuring an efficient and safe glucose control by detecting fluctuations in glucose levels and giving a precise picture of what a patient’s condition is (5, 6). These devices are crucial especially for patients with high risk of hypo- or hyperglycemia. Diabetes Monitoring Systems (DMS) are systems which integrate one or more GMS devices to support diabetes management. On the basis of the literature, currently available different types of DMS could be classified in several groups: conventional (glucose meters), continuous glucose monitoring systems (CGMS) being a variety of devices (professional, personal, retrospective, real-time, flash, etc.), non-invasive, closed-loop systems, Sensor Augmented Pump Therapy (SAPT), and telemedicine/mobile technologies which integrate glucose monitoring systems or telemonitoring (**Figure 1**). The conventional ones are commonly used by patients but the small number of measurements per day causes the unawareness of fluctuations in glucose levels and asymptomatic hypoglycemia. Continuous glucose monitoring systems measure glucose levels continuously throughout the day and provide information on the glucose values fluctuations (7, 8). The main advantage of closed-loop systems (so-called artificial pancreas systems) and sensor augmented pump therapy is the option to allow precise adjustment of patient’s insulin injections due to transmission of glucose readings between CGMS and insulin pump. These combined devices lead to increased life expectancy, delayed onset and prоgression of microvascular complications as they are an effective method for improvement of metabolic control (9–11). Further development of more sophisticated glucose-monitoring devices and techniques could help to overcome many challenges such as reduction of pain due to frequent pricking of skin for the purposes of glucose levels testing (1). Telemedicine systems or mobile applications which integrate glucose monitoring systems have shown an increasing adoption to improve adherence to treatment and contribute to improve diabetes management.

Many systematic and literature reviews focusing on effectiveness of glucose monitoring devices are published in the literature. They differ in terms of the type of analyzed glucose monitoring device, type of diabetes, type of study—systematic review or systematic review and meta-analysis. No studies gather and systematize the available evidences for all diabetes patient subgroups on use of all types of DMS, their application, effectiveness, and cost-effectiveness. Due to expanding knowledge and inconsistent results between published studies we attempted to perform a systematic review of already published systematic reviews and systematic reviews + meta-analysis. Moreover, the future in diabetic patients’ medical care is in finding the most suitable individualized approach for treatment, diagnosis, or monitoring condition for the purposes of achieving the desired outcomes. Therefore, a comprehensive patient-oriented analysis of the available diabetes monitoring systems is required which could be used as a basis for defining the effective and cost-effective approaches for regular monitoring and control of diabetes patients. Furthermore, as an ever-growing body of evidence emerges, new ways of agglomerating all available data will be needed, in order to consolidate all relevant information to help decision makers paint a clearer picture. Our attempt in this paper to use existing methods in a different way could provide a steppingstone upon which to build more reliable assessments in diabetes. This work is part of the H2020 HTx project, whose goal is to provide a new generation of health technology assessments (https://www.htx-h2020.eu/). For this purpose, the project proposes to apply technological improvements to data curation, combining evidence extracted from real-world data sources in addition to evidence obtained reviewing existing methods.

The primary goal of the study is to review and consolidate evidences from multiple systematic reviews on the diabetes monitoring systems and the outcomes achieved. In addition, we wanted to systematize the approaches used for personalized treatment and monitoring of diabetes patients *via* new technologies. Moreover, this paper’s results and conclusions will be used as a basis for development of future technological improvements in the HTx project.

## Materials and Methods

### Study Design

A comprehensive systematic review of published systematic reviews investigating the effect of diabetes monitoring systems was performed. It was based on the following approach: (1) identification of a research question; (2) identification of inclusion and exclusion criteria; (3) data extraction; (4) reporting results; (5) assessment of risk of bias; (6) discussion and interpretation of the results. The research questions formulated during the study were focused on the available new technologies for diabetes monitoring; available systematic reviews and meta-analyses reporting the treatment outcomes; types of therapeutic outcomes reported and their variability.

The type of treatment outcomes that were observed were changes in the clinical parameters (HbA1c, glucose level, etc.); changes in the quality of life (QoL); number and frequency of hypo- and hyperglycemia episodes; change in the risk for complications; level of satisfaction from the intervention.For the identification of the studies, we performed an internet search of electronic databases PubMed, Cochrane library, Embase and proposed by them related articles. The key words for the search were “diabetes” AND “systematic review,” AND “CGMS” OR “SAPT” OR “CSII” OR “FGM” OR “Closed loop systems” OR “telemedicine.” For the presentation of the search results was used the principles of the PRISMA checklist with its four steps the search approach, as follows: identification, screening, eligibility, and inclusion. The search encompasses the period since the first systematic review identified till the end of 2019.

### Inclusion and Exclusion Criteria

Specific inclusion and exclusion criteria were formulated. The criteria for inclusion were to be a systematic review of interventional or non-interventional (observational) studies about diabetes monitoring systems, to present clinical and/or economic effect of new technologies on diabetes patients, English language. The criteria for exclusion were lifestyle maintaining technologies, medicines, diet,exercise, algorithms’ decision supporting systems, mobile apps (especially those that relate to the lifestyle maintaining), alarms, and m-health. Our focus on excluding some studies was more on removing ones that rely solely on telemonitoring by physicians. If more than two studies were found from the same authors, the latest published article was taken into account.

### Selection of Studies

Five authors (MK, MD, ZM, KT, GP) reviewed selected articles for duplication, relevance to the inclusion and exclusion criteria, and consolidate the evidences by systematizing them according to the technology described, type of the diabetes for which the technology is recommended, sources of information, clinical and/or economic results reported, recommendations for future application or improvement. Each author independently reviewed the articles for eligibility. Discrepancies between the authors were overcome through discussion, until reaching consensus. The main focus was on technologies aiming to improve diabetes control *via* improvement in glycated hemoglobin (HbA1c), hypo- or hyperglycemic episodes, or glucose secretion monitoring and not on improving the lifestyle habits and compliance.

### Data Extraction and Summarization

The data extracted were summarized in tables including the following relevant information:

1. **Supplementary Table 1**—excluded full-text articles with reasons, publication year, name of the first author, type of technology observed, type of diabetes, type of review (systematic review or systematic review and meta-analysis), search strategy (database searched), number of relevant studies, and total number of participants;
2. **Table 1**, **Table 2**, and **Table 3**—included studies, reasons for inclusion, publication year, name of the first author, type of technology observed, type of diabetes, type of review (systematic review or systematic review and meta-analysis), search strategy (database searched), number of relevant studies, and total number of participants; intervention observed, comparator(s), change in clinical parameters, change in quality of life; comments and strength of evidence.

**Table 1:** Characteristics of the included systematic reviews and meta-analyses.

Review, year	Technology	Type of diabetes	Type of review	Search strategy (database searched)	Number and design of included studies	Total number of participants
Smith MB et al. (12)	CGM	T1DM	Systematic review	PubMed, CINAHL, the Cochrane Library, PsychInfo database	26 (cross-sectional; observational; Uncontrolled pre, post intervention; RCT: open-label, crossover RCT;	7377
Cowart K et al. (13)	CGM (flash GM)	T1DM or T2DM	Systematic review	PubMed, EMBASE, Cochrane Library	9 RCTs	1064
Voorlmolen DN et al. (14)	CGM	T1DM, T2DM pregestational and gestational diabetes	Systematic review	PubMed, EMBASE, Cochrane Library	11 (only 2 of them are RCTs)	539
Park C et al. (15)	CGM (RT- CGM and professional CGM)	T2DM	Systematic review and meta-analysis	Cochrane, EMBASE, PubMed, Web of Science	7 RCTs and 3 cohort studies	6286
Karageorgiou V et al. (16)	Artificial pancreas (closed-loop system)	T1DM	Systematic review and meta-analysis	Medline, Scopus, Cochrane Central Register of Controlled trials, Clinicaltrials.gov, Google Scholar	25 RCTs, 19 of which are included in the meta-analysis	504
Weisman A et al. (17)	Artificial pancreas (closed-loop system)	T1DM	Systematic review and meta-analysis	Medline, Embae, Cochrane Central Register of Controlled Trials	24 studies—23 crossover and 1 parallel	585
Poolsup N (18)	CGM and SMBG	T1DM pediatrics and T T2DM adults	Systematic review and meta-analysis	MEDLINE (pubmed), SCOPUS, CINAHL, Web of Science,The Cochrane Library	14 RCTs	817 pediatrics; 161 adult
Garcia-Lorenzo et al. (19)	[RT-CGM] *vs.* [SMBG]	T1DM T2DM	Systematic review and meta-analyses; cost-effectiveness analysis using a Markov model	MEDLINE, PreMEDLINE, Cochrane Central Register of Controlled Trials, and Social Science Citation Index	17 RCTs	1 843
Golden et al. (20)	[RT-CGM] *vs.* [SMBG]	T1DM	Systematic review and meta-analyses	MEDLINE^®^, Embase^®^, and the Cochrane Central Register of Controlled Trials	9 RCTs	1 246
	[SAPT] *vs.* [MDI*/SMBG]				4 RCTs	612
Jones et al. (21)	CGM *vs.* SMBG	T1DM, T2DM pregnant women	Systematic review	Cochrane Pregnancy and Childbirth’s Trials Register, ClinicalTrials.gov, the WHO International Clinical Trials Registry Platform (ICTRP)	12 RCTs and quasi‐RCTs	944
Mattishent K, (22)	[CGM] *vs.* [SMBG]; [CGM] *vs.* [no CGM] [RT-CGM] *vs.* [no RT-CGM]	T1DM or T2DM >/=65 years	Systematic review	SCI Web of Science, Ovid SP MEDLINE and EMBASE	9 (RCTs; observational trials)	989
Moy F et al. (23)	[SMBG] *vs.* standard care	T1DM or T2DM pregnant women	Systematic review and meta-analyses	Cochrane Pregnancy and Childbirth Group’s Trials Register	9 RCTs and quasi‐RCTs	506
Waite et al. (24)	[Telemedicine system + insulin pump/RT-CGM] *vs.* [insulin pump/RT-CGM at baseline]; [Automated telemedicine] *vs.* [conventional system]	T1DM	Systematic review	Computing Research Repository; PsycINFO, EMBASE, and MEDLINE; Web of Science; Zetoc; Excerpta Medica and Scopus; and ProQuest.	18 (observational; RCT; cross-sectional, qualitative)	3 320
Medical Advisory Secretariat (25)	[Home telemonitoring] *vs.* [usual (routine) SMBG]	T2DM	Systematic review and meta-analysis	OVID MEDLINE, MEDLINE In-Process and Other Non-Indexed Citations, EMBASE, the Cumulative Index to Nursing & Allied Health Literature (CINAHL), The Cochrane Library, and the International Agency for Health Technology Assessment (INAHTA)	8 (RCTs, surveillance, case series, retrospective review, modeling)	2 269
Hsin-Chieh Yeh (26)	Rapid acting analogues based CSII; RT-CGM; SAPT	T1DM and T2DM	Systematic review and meta-analysis	MEDLINE, EMBASE, and the Cochrane Central Register of Controlled Trials through February 2012 without languagerestrictions.	33 RCT	Not stated
Szypowska (27)	Rt-CGM *vs* SMBG	T1DM	Systematic review and meta-analysis	MEDLINE, EMBASE, and the Cochrane Library from 1996 to March 2011.	7 RCT	948
Bidonde J (28)	FreeStyle Libre Flash Glucose Self-Monitoring System	T1DM and T2DM	Systematic review and cost-effectiveness analysis	Databases, trial registries, health technology assessment agencies websites, and gray literature from inception to January 2017. No language restrictions	2 RCT	463
Langedam M (29)	CGM compared to SMBG	T1DM	Systematic review	The Cochrane Library, MEDLINE, EMBASE, and CINAHL	22 RCT	2243
Raman R (30)	CGM *vs.* SMBG	Gestational diabetes	Systematic review	Cochrane Pregnancy and Childbirth Group Trials Register	11 RCT	1272
Hill S (31)	[CSII] *vs.* multiple daily injections [MDI] and/or real time-continuous glucose monitoring [RT-CGM] *vs.* self monitoring of blood glucose [SMBG]	T1DM, T2DM, and preexisting diabetes in pregnancy	Systematic review	MEDLINE, Embase, Cochrane Central Register of Controlled Trials	41 RCT and observational studies	4393
Dai Xia (32)	Closed-loop system *vs* control group	T1DM	Systematic review and meta-analysis	Medline database, the Cochrane library, EMBASE	8 RCT	354
De Ridder F (33)	[CGM (FGM and RT-CGM) and insulin delivery from MDI, *via* SAPT and (predictive) low-glucose insulin suspension to hybrid closed-loop systems]	T1DM	Systematic review	PubMed and the Cochrane library up to 30 May 2019	19 RCT	1450
Wojciechowski P (34)	CGM *vs* SMBG	T1DM	Systematic review and meta-analysis	MEDLINE, EMBASE, CENTRAL, Trip Database, and the Centre for Reviews and Dissemination	14 RCT	1268
Yeoh E (35)	New technologies	T1DM	Systematic review and meta-analysis	We searched The Cochrane Library, MEDLINE, Embase, Science Citation IndexExpanded, Social Sciences Citation Index, PsycINFO, and CINAHL	43 studies (11 technological)	Not stated
Yu Q (36)	CGM and SMBG	Gestational diabetes	Systematic review	PubMed, Scopus, and Web of Science	29 articles (3 RCTs; 1 randomized crossover trial, 25 prospective observational cohorts)	1717
Golicki (37)	[Continuous Glucose Monitoring] *vs* [self-monitoring glucose]	T1DM	Systematic review and meta-analysis	1966–2007 MEDLINE, EMBASE, and The Cochrane Library of randomized controlled trial	5 RCTs	70
Hill-Golden S (38)	[Continuous subcutaneous insulin infusion (CSII) *vs.* multiple daily injections (MDI)]^a^ and/or [real time-continuousglucose monitoring (rt-CGM) with self-monitoring of blood glucose (SMBG)]^b^; [Sensor- augmented pumps *vs* MDI and SMBG]^c^	T1DM and T2DM	Comparative effectiveness review of previously published systematic review (26), and its expansion (40) stratified by age	MEDLINE, EMBASE, and the Cochrane Central Register of Controlled Trials. 1994–2011	Total 44 studies a) 28 [9 children with DMT1]; [9 Adults with DMT1]; [4 studies, 5 publications for Adults with T2DM]; [6 for pregnant women with pre-existing T1DM and T2DM] b) 9 studies, 10 publications: [9 studies, 10 publications for children and adults with type 1] c) 4 studies, 5 publications for children and adults with T1DM	Children and adolescents with T1DM – NR Adults with T1DM – NR Adults with T2DM – 20 to 66 Pregnant women with pre-existing - NR
Gandhi G et al. (2011) (41)	[Continuous glucose monitoring (CGM)] *vs* [self-monitored blood glucose (SMBG)]	T1DM or T2DM	Systematic Review	MEDLINE, EMBASE, Cochrane Central, Web of Science, and Scopus	19 RCTs	1801
Bandeira-Echler et al. IQWiG Reports (2015) (42)	[Continuous interstitial glucose monitoring (CGM) with real-time measurement devices] *vs.* [blood glucose self-monitoring (BGSM)], [retrospective CGM], and [variants of rtCGM]	T1DM	Systematic Review and Meta-Analysis			
Hoeks et al. (2011) (43)	[Continuous Real-time Glucose Monitoring system] (rt-CGSM) *vs.* non-real-time Continuous Glucose monitoring system (CGSM)] or self-monitoring blood glucose	T1DM and T2DM	Systematic Review	PubMed/MEDLINE and EMBASE from 01.2005 to 01.2010	9 RCTs	NR
Janapala et al. (2019) (44)	[Continuous Glucose Monitoring systems (CGSM)] *vs.* [Self-monitoring of Blood Glucose (SMBG)]	T2DM	Systematic Review and Meta-Analysis	PubMed/Medline	20 (5 RCTs)	From the 5 RCts - 374

*RCTs, randomized clinical trials; T1DM, type 1 diabetes mellitus; T2DM, type 2 diabetes mellitus.

**Table 2:** Key findings of the included systematic reviews and meta-analyses by type of intervention.

Intervention observed(Type of study)	Identified systematic reviews	Comparator	Type of diabetes	Change in clinical parameter (HbA1c, glucose levels, etc.)	Change in QoL	Hypo-hyper glycemia	Change in the risk for complications	Level of satisfaction from the intervention
SMBG*								
Meta-analysis	Moy FM** (23)	Standard care	T1DM or T2DM pregnant women	0.1% [−1·87–1·67] reduction in HbA1c; 0.7% [−2·15–0·75] reduction in maternal post-prandial blood glucose				
CGM*								
Systematic review	Mattishent (22)	no CGM	T1DM (≥65 or older)	0·5%, (P <·0001) reduction in HbA1c		- 0.25 less SH per year, P = ·0007.		
		RT-CGM *vs.* no RT-CGM				- 7 less severe hypoglycemia episodes per week		RT-CGM improves feelings safety and well-being
		SMBG	T1DM or T2DM (≥65 or older)	0·4 ± 0·1%, P < ·001 reduction in HbA1c				
		RT-CGM *vs.* no RT-CGM			Well-being: 3·3 *vs.* 2·7 (P < ·001); Hypoglycemic fear: 27·1 *vs.* 31·5 (P < ·05); Overall diabetes distress: 2·2 *vs.* 2·5 (P < ·05)			
	Smith (12)	SAP/ SMBG/ No comparator	T1DM	No significant change in the values of HbA1c compared to SMBG	No significant improvements in general HRQoL	Reduction in time in hypoglycemia compared to SMBG		Greater satisfaction related to device accuracy (P < 0·05) and ease of use (P < 0·01)
	Golicki (37)	SMBG		No reduction in HbA1c (WMD −0·02%, 95% CI −0·29 to 0·25; p = 0·87)	NR	No major hypoglycemic events. Non-significant difference for minor hypoglycemic episodes (mean difference 0·53, 95% CI −0·68 to 1·74; p = 0·39)	Minor skin and site reactions.	
	Jones (21)	intermittent glucose monitoring	T1DM or T2DM pregnant women pregnant women with either preexisting DM or GDM			- Reduction of hypertensive disorders: RR = 0·58, 95% CI 0·39–0·85); - Reduction of neonatal hypoglycemia: RR = 0·66, 95% CI 0·48–0·93.		
	Voormolen (14)	SMBG		No significant change in HbA1c during 1st and 2nd trimester; Significant difference (p = 0·007) in 3rd trimester				
	Cowart (13)	SMBG/CSII	T1DM and T2DM	Reported change in the values of HbA1c ranging from −0·5 to 0·17% (*post-hoc*) compared to SMBG		Reduction in time in hypoglycemia compared to SMBG and reduced risk by 54% for nocturnal hypoglycemia		Greater satisfaction compared to SMBG
	Langendam (29)	CGM *vs* SMBG	T1DM	HbA1c level −0·7%, 95% confidence interval (CI) −0·8 to 0·5% HbA1c level −0·2%, 95% CI −0·4 to −0·1% for new users	No significant difference between CGM and SMBG	RR of hypo increased 4/43 *versus* 1/35; RR 3·26, 95% CI 0·38 to 27·82 and 21/247 *versus* 17/248; RR	Ketoacidosis after 6 months—one study	
	Raman (30)	CGM *vs* versus self-monitoring of glucose	Gestational diabetes	−0·10%, 95% CI −0·24 to 0·04			Cesarean section (RR 0·91); - neonatal hypoglycemia (RR 0·79). There were no perinatal deaths.	
	Hill (31)	RT-CGM *versus* SMBG	T1DM	Rt-CGM favored over SMBG—Mean between-group difference in HbA1c from baseline was −0·30% (95% CI, −0·37 to −0·22%).	Diabetes-specific QOL did not differ between the rt-CGM and SMBG arms (mean between-group difference in Problem Areas in Diabetes score, −0·9;95% CI, −7·9 to 6·1 at 26 weeks,73 and mean between-group difference in the change from baseline Diabetes Quality of Life score, −3·0; 95% CI, −6·6 to 0·665).	Severe hypoglycemia did not differ between the rt-CGM and SMBG groups (pooled RR, 0·95; 95% CI, 0·53 to 1·69). Significant reduction in time spent in the hyperglycemic range—mean difference of −68·56 min/day favoring rt-CGM (95% CI, −101·17 to −35·96).	None of the studies evaluated the effects of rt-CGM *vs.* SMBG in terms of mortality, microvascular or macrovascular disease, weight, or any other process measure.	60% satisfaction
	De Ridder Fr. (33)	Rt-CGM *vs* isCGM	T1DM	No difference in HbA1c Time in range (7·4 to 4·7% difference)	Fear of hypo	−4·3% in favor of RT-CGM		
	De Ridder Fr. (33)	Rt-CGM *vs* SMBG	T1DM	Insignificant decrease in HbA1c level (−0·43 to −0·47%)		Time in hypo: Rt-CGM: 0·4–0·1h/day SMBG: 0·65–0·6 h/day		High satisfaction
	Yu Q (36)	CGM and SMBG	Gestational diabetes			CGM detected more hypoglycemia and hyperglycemia incidents	Compared with SMBG, CGM users have lower incidence of preeclampsia [5 out of 150 (3·3%) *vs.* 19 out of 190 (10%), P = 0·019], primary cesarean section out of 150 (34·0%) *vs.* 88 out of 190 (46·3%), P = 0·028], and premature delivery [7 out of 150 (4·7%) *vs.* 22 out of 190 (11·6%), P = 0·024]	Most patients felt that CGM is easy to use (44 out of 48, 92%), beneficial for self-glycemic control (43 out of 48, 90%), and that its use outweighed its inconvenience (37 out of 48, 77%)
Meta-analysis	Garcia-Lorenzo (19)	RT-CGM *vs.* SMBG	T1DM	WMD* = −0·23% *(95% CI: −0*·*35, −0*·*11);*	ΔQALY = 0·046	No difference*: OR = 1*·*16 (95% CI: -0*·*79, -0*·*17)*		
	Golden (20)	RT-CGM *vs.* SMBG		0·30–0·36% reduction in HbA1c				
	Poolsup (18)	SMBG		retrospective CGM was not superior MD-0·05% (95%CI −0·46 to 0·35%)] RT-CGM revealed better effect [MD-0·18% (95% CI −0·35 to 0·02%, p = 0·02)].				
	Hill-Golden S (38)	SMBG		Children and adolescents: −0·26 (−0·46, −0·06) (p = 0·248) Adults: −0·30 (−0·30, −0·22) (p = 0·04) Adults with compliance >60% −0·36 (−0·44, −0·27) (p = 0·119)				
	Moy FM** (23)	intermittent glucose monitoring	T1DM or T2DM pregnant women	0·34% [−0·83, 0·15] reduction in HbA1c;		- RR = 0·77 [0·51, 1·17] for neonatal hypoglycemia		
	Garcia-Lorenzo (19)	RT-CGM *vs.* SMBG	T1DM	WMD* = −0·48% *(95% CI: −0*·*79, −0*·*17)*	ΔQALY = 0·272			
	Park C (15)	SMBG		0·20% reduction in HbA1c		Significant reduction in hypoglycemia compared to SMBG		
	Poolsup N (18)	SMBG		reduction in HbA1c with CGM [MD – 0·31% (95% CI −0·6 to −0·02%, p = 0·04)]				
	Janapala R.N (44)	SMBG		The pooled mean difference in HbA1c was −0·25 (−0·45, −0·06) and statistically significant (at p = 0·01) whencomparing CGM to SMBG.	Some studies have shown that CGM data did not differ significantly from the controls, which may be explained by the fact that these populations could be relatively healthy with lesser glycemic excursions. Therefore, these studies have insufficient power in detecting a significant difference between the groups			One study reported very high compliance with CGM usage; 97% of the subjects used it for 6 or more days per week for 6 months. A satisfaction survey indicated very high satisfaction with CGM.
	Hsin-Chieh Yeh (26)	Rt-CGM *vs* SMBG	T1DM	rt-CGM reduced HbA1c levels more than SMBG heterogeneous results	No difference	No difference		
	Szypowska (27)	Rt-CGM *vs* SMGB	T1DM	HbA1c −0·25; (95% CI: from −0·34 to −0·17; P 0·001)	Not evaluated	No influence on major hypoglycemic incidents (six RCTs, nZ864, RR 0·69; 95% CI: 0·41–1·14; PZ0·15).) Difference in hyperglycemia in favor of RT-CGM		The compliancewith the sensor wear was age related and lower in children and the lowest in adolescents
	De Ridder Fr. (33)	isCGM *vs* SMBG or CGM.	T1DM	Hb1Ac did not change significantly (−0·43 to −0·36) over 6 months Time in range increased significantly (4·2–6·5%).	Improved QoL	Time in hypoglycemia decreased significantly (3·38 to 0·75 h/day)		Improved treatment satisfaction
	Wojciechowski P (34)	CGM *vs* SMBG	T1DM	Patients using CGM had a greater decrease in HbA1c from baseline compared with those using SMBG (WMD –0·26% [–0·34; –0·19]). Only real−time devices for CGM improved glycemic control (WMD –0·27% [–0·34; –0·19]).		Reduction in hypoglycemic events in the CGM group (SMD –0·32 [–0·52; –0·13]). Significant reduction of hypoglycemic events in the CGM group *vs* SMBG group (SMD –0·32 [–0·52; –0·13]).		
	Yeoh (35)		T1DM	CGM reduced severe hypoglycemia, improved glycemic control, and restored awareness in combination with structured education and frequent contact.				
	Yeh H.C (40)	SMBG	T1DM and T2DM	All studies −0·26 (−0·33 to −0·19) Adults >18 y −0·38 (−0·53 to −0·23) Children <18 y −0·13 (−0·27 to −0·01) Adherence >60% −0·36 (−0·44 to −0·27)	Although QOL was measured by using different instruments, all studies reported no difference between groups	No difference in time spent in the hypoglycemic range; Significant reduction in time spent in the hyperglycemic range with a mean between-group difference of 68·56 min/day (CI, 101·17 to 35·96 min/d)		.
	Yeh H.C (40)	SMBG	T1DM and T2DM	(WMD) of −0·27% (95% CI −0·44 to −0·10). For adults with T1DM as well as T2DM: WMD −0·50% (95% CI −0·69 to −0·30) and −0·70 (95% CI, −1·14 to −0·27), respectively. Non-significant changes in children and adolescents No significant difference in HbA1c reduction between studies of real-time *versus* non-real-time devices (WMD −0·22%, 95% CI, −0·59 to 0·15 *versus* −0·30%, 95% CI, −0·49 to −0·10; p for interaction 0·71).	Quality-of-life measures did not change with the use of CGM.	RR for hypoglycemia = 1·02 (95% CI, 0·3 to 3·45). Using the number of events as the unit of analysis, the rate ratio for hypoglycemia was 3·50 (95% CI, 1·07 to 11·44) and for hyperglycemia 1·42 (95% CI, 0·26 to 7·82)		Some participants stopped wearing the continuous glucose sensors because of inconvenience, problems sleeping, bathing, and difficulty taking part in sporting activities
	IQWiG Reports (42)	SMBG only		There were statistically significant differences between the treatment options regarding patient-relevant outcomes only for the comparison of rtCGM plus BGSM *versus* BGSM.	In the comparison of rtCGM plus SMBG *versus* SMBG, there were statistically significant differences only regarding the joint consideration of severe or serious hypoglycemia and HbA1c value, skin reactions, and individual instruments or subscales of health-related quality of life.		Evaluable results on skin reactions were reported in one study.	SMBG only
	Gandhi G (41) Hoeks L.B (43)	SBMG and / or the offline continuous glucose monitoring system	T1DM and T2DM	6 studies: positive effect (0·3–0·7% or 3–8 mmol/mol) of the real-time continuous glucose monitoring system on HbA1c compared with the control; 3 trials: increased HbA1c improvement in patients with better compliance; 1 study: HbA1c was 0·51% lower in participants who wore the sensor ‡ 70% of the total study period (98% CI 0·04–0·98%, P = 0·04) Another study showed that each 10% increase of time the sensor was used was associated with a 41% increase in the probability of a 0·5% reduction in HbA1c	Quality of life was not assessed in any of the studies.	None of the seven studies demonstrated a positive effect of the real-time continuous glucose monitoring system on the incidence of severe hypoglycemia.		
CLOSED-LOOP SYSTEMS (ARTIFICIAL PANCREAS)								
Мeta-analyses	Karageorgiou (16)	CSII	T1DM	Significantly increased % of time in target glycemic range (MD: −11·97%, 95% CI)		Significant decrease in time in hypo-and hyperglycemia (MD 0·67 and 3·01%, respectively)		
	Weisman (17)	CSII + CGM or CSII + SAP		Significantly increased % of time in target glycemic range (MD: −12·59%, 95% CI) compared to CSII		Significant decrease in time in hypo- (MD 2·45%)		
	Dai Xia (32)	Artificial pancreas *vs* control group	T1DM	Maintain a better mean concentration of glucose (WMD −1·03, 95% CI −1·32 to −0·75; P = 0·00001).		Time spent in the hypoglycemic phase is significantly lower (WMD −1·23, 95% CI −1·56 to −0·91; P = 0·00001). The numbers of hypoglycemic events were not significantly different.		
SAPT*								
Meta-analysis	Golden SH (31)	SAPT *vs.* MDI*/SMBG	T1DM	0·61% reduction in HbA1c				
	Yeh (26)	SAP *vs.* MDI or SMBG	T1DM	−0·68% reduction in HbA1c	Insufficient evidence	Hyperglycemia significantly shorter with SAP than with MDI or SMBG (P > 0·001). Insufficient evidence for hypoglycemia		
Systematic reviews	Yeh et al. (26)	SMBG	T1DM and T2DM	SAPT decreased HbA1c levels more than MDI or SMBG did (combined mean between-group difference from baseline,0·68%)		The time spent with hyperglycemia was significantly shorter with the SAP than with MDI or SMBG (P = 0·001). Severe hypoglycemia occurred at a similar rate in the SAP and MDI or SMBG groups (21 out of 247 *vs.* 17 out of 248; P = 0·58) with a risk difference of 1·6% (CI, −3·0% to 6·3%).		
TELEMONITORING								
Systematic review	Waite M et al. (24)	iOS app—Glucose Buddy—combined with text messaging feedback *vs.* no intervention	T1DM	1·10%, SD = 0·74 (P </= 0·001) reduction in HbA1c;				
		Telemedicine system +insulin pump/RT-CGM *vs.* insulin pump/RT-CGM at baseline		0·53%, P = 0·01 reduction in HbA1c; 15·6, P = 0·04 reduction in glucose variability	5·5 scores, P = 0·01 improvement in quality of life			2·7, P = 0·01 improvement in satisfaction
Meta-analysis	Moy FM** (23)	Automated telemedicine *vs.* conventional system	T1DM or T2DM pregnant women	0·35% [−1·13, 0·43] reduction in HbA1c; 0·8% reduction in maternal post-prandial blood glucose				
	Medical Advisory Secretariat (25)	Home telemonitoring *vs.* usual SMBG	T2DM	0·5% reduction in HbA1c (statistically significant)				
CSII								
Systematic review and meta-analysis	Yeh (26)	CSII *vs* multiple daily injections [MDI]	T1DM and T2DM	Adult T1DM (HbA1c −0·30% from −0·58 to −0·002) Adult T2DM (no difference in mean decrease of HbA1c (−0·18%) Children T1DM—no difference in meta-analysis and RCTs	Adult T1DM improved diabetes mellitus–specific QOL favoring CSII Adult T2DM—insufficient evidences for QoL difference Children T1DMfavor CSII	Adult T1DM—Mixed results not in favor of CSII Adult T2DM—insufficient evidences about the effects on nocturnal hypoglycemia, hyperglycemia, Children T1DM—Severe hypo no difference		Children T1DM—favor CSII
Systematic review	Hill S. (20)	[CSII] *vs.* multiple daily injections [MDI]	T1DM, T2DM and preexisting diabetes in pregnancy	T2DM—no difference in HbA1c between-groups from baseline with negative value favoring CSII, −0·16; 95% CI, −0·42 to 0·09). T1DM—decreased more with CSII than with MDI (mean between-group difference from baseline, −0·30%; 95% CI, −0·58 to −0·02 or −0·01%). Preexisting T1DM diabetes during pregnancy—improvement in HbA1c in both the CSII and MDI groups during pregnancy without any significant difference between groups—mean difference 0·2 (95% CI, −0·3 to 0·7), −0·4 (95% CI, −0·8 to 0·04), 0·6 (95% CI, −0·7 to 1·9); −0·3 (95% CI, −0·6 to −0·03), 0·2 (95% CI, −0·2 to 0·6), and 0·4 (95% CI, −0·9 to 1·7)	T2DM—No difference in general QOL between the CSII and MDI intervention groups. The difference from baseline to follow-up was 0·6 for CSII *vs.* 0·4 for MDI for the SF-36v2 Physical Component Score, and 1·0 for CSII *vs.* 2·5 for MDI for the Mental Component Score T1DM—improvement in general QOL between the two intervention groups favoring CSII	T2DM Risk of severe hypoglycemia did not differ between CSII and MDI (RR, 0·76; 95% CI, 0·26 to2·19). Hyper—the incidence of blood glucose over 350 mg/dl was higher in the MDI than CSII arm (26 *vs.* 6 events), affecting 18 and 5% of participants in the MDI and CSII arms, respectively (RR, 0·28; 95% CI, 0·08 to 0·94). T1DM—nocturnal hypoglycemia was similar in the MDI and CSII; increased risk of symptomatic hypoglycemia for CSII compared with MDI (combined IRR, 1·3; 95% CI, 1·2 to 1·4).	T2DM—Did not identify any studies evaluating the effects of MDI *vs.* CSII among patients with T2DM in terms of any of the micro-vascular or macro-vascular disease. T1DM—not measured Preexisting T1DM diabetes during pregnancy—for major congenital anomalies a pooled RR of 2·12 favoring MDI (95% CI, 0·38 to 11·77)—inconclusive because of high risk of bias.	T2DM improvement in diabetes treatment satisfaction favoring CSII (mean between-group difference change from baseline in 24 weeks, 13·1; 95% CI, 7·4 to 18·8) T1DM—not measured
Sytematic review and meta-analysis	Wojciechowski (34)	CSII *vs* MDI	T1DM	Improvement of HbA1c of 0·5%.				
	Wojciechowski (34)	Rt-CGM *vs* SMBG	T1DM	Not stated		Improvement in hypoglycemia awareness		
Systematic review	Hill S. (20)	*rt-CGM + CSII) Versus MDI/SMBG*	T1DM	HbA1c - sensor-augmented pumps favored over MDI/SMBG (mean between group difference in HbA1c change, -0·68%; 95% CI, -0·81 to -0·54%).	Blood Glucose Monitoring System Rating Questionnaire scores were 83·3 ± 21·7 for sensor-augmented pump *vs.* 33·3 ± 22·6 for MDI/SMBG (mean between-group difference in final scores, 50·0; 95% CI, 33·6 to 66·4)	Hyperglycemia was significantly less in the sensor-augmented pump group than the MDI/SMBG intervention group (P < 0·001). Severe hypoglycemia did not differ.	Not evaluated	User acceptance and overall diabetes treatment satisfaction greater in the sensor-augmented pump than the MDI/SMBG arm.
FREESTYLE LIBRE SYSTEM—A “WIRELESS” METHOD USING A SENSOR FOR MONITORING INTERSTITIAL FLUID GLUCOSE								
Systematic review and cost-effectiveness analysis	Bidonde (28)	FreeStyle Libre Flash Glucose Self-Monitoring System *vs* SMBG and European assessment	T1DM and T2DM	−0·00 (95% CI ‐0·14 to 0·14; I2 = 0% indicating no heterogeneity; P = 0·81)	Mean difference −0·05 (95% CI −0·16 to 0·05; I2 0% indicating no heterogeneity; p = 0·36)	Hypoglycemia −0·23 (95% CI −0·35 to −0·10; I2 = 64% indicating substantial heterogeneity; p = 0·09). time spent in hypoglycemia −0.22 (95% CI −0·46 to 0·03)		5·10 (95% CI 2·95 to 7·26; I2 = 70% indicating substantial heterogeneity; P = 0·07) with Diabetic treatment satisfaction questionnare

*CGM, Continuous Glucose Monitoring; DM, diabetes mellitus; GDM, gestational diabetes mellitus; MDI, Multiple Daily Injections; SAPT(SAP), Sensor Augmented Pump Therapy; SH, severe hypoglycemia; SMBG, Self-monitoring Blood Glucose; QALY, Quality Adjusted Life Years; WMD, Weighted Mean difference in HbA1c levels; CSII, continuous subcutaneous insulin infusion. **{NO SIGNIFICANT ADVANTAGES} There is no evidence that any glucose monitoring technique is superior to any other technique among pregnant women with pre-existing Type 1 or Type 2 diabetes. Additional evidence from large well-designed randomized trials is required; T2DM, type 2 diabetes mellitus; T1DM, type 1 diabetes mellitus.

**Table 3:** Summary of the main results and conclusions.

Type of intervention	Number of positive assessments	Number of negative assessments	Overall conclusions	Comments	Strength of Evidence
Self-Monitoring Blood Glucose	1	0	Moderate to high reduction in HbA1c and post-prandial blood glucose. Conclusions are consistent with results from individual studies	It is well established that frequent self-monitoring improves outcomes and control compared to standard care	The included analysis is of moderate Quality (45)
Continuous Glucose Monitoring	22	6	Most studies observed a mild reduction in HbA1c. However, moderate evidence suggests it reduces complications (hypo-hyperglycemia)	It appears that in patients with low compliance, CGM systems are effective, but not so much in patients with high compliance	GRADE 1 study—very low (46) 2 studies—low (25, 39) 9 studies—moderate (12, 13, 16, 18, 39, 45, 47, 48) 16 studies—high (13, 15, 17, 49–61)
Telemonitoring	3	0	Telemonitoring devices and approaches give a statistically significant reduction in HbA1c and more time spent in range.	One study included patients who are on CGM and pump systems and did not differentiate well if the observed reduction was due to the system or the telemonitoring. One of the studies had “no intervention” as a comparator, which skews the results.	1 study—very low (62) 1 study—low (63) 1 study—moderate (45)
Sensor-Augmented Pump Therapy	2	0	Consistent reduction in HbA1c, reduction in time spent in hyperglycemia.	There is insufficient evidence on quality of life and patients’ acceptance and adherence to technology	2 studies of high quality (57, 58)
Closed-Loop System (artificial Pancreas)	3	0	Significantly increases time in HbA1c Range in T1DM. Additionally, decreases hypoglycemia incidents and time spent in hypoglycemia	Offer extremely high and reliable therapy	1 study—low (64) 1 study—moderate (65) 1 study—high (66)
FreeStyle Libre System ‐ Flash Glucose Monitoring	1	0	No effect on HbA1c. Non-significant reduction in time spent in hypoglycemia.	No-comments. The evidence is insufficient to adequately comment on technology	Very low quality (67)

### Data Analysis

The upper and lower changes in clinical parameters were summarized by intervention, type of diabetes, and type of review [systematic review (SR) or systematic review and meta-analysis (SR/MA)]. The results were presented in tables and figures. We have not included detailed information about the sample size of primary studies and location of the study because we have focused mainly on the clinical outcomes described in the published systematic reviews and/or meta-analyses.

### Assessment of the Risk of Bias in the Included Systematic Reviews

The methodology quality of systematic reviews and meta-analysis included in the current systematic review was assessed using the GRADE system (**Table 3**). GRADE has four levels of evidence—also known as certainty in evidence or quality of evidence: very low, low, moderate, and high. All included SRs and MAs were evaluated in five domains—Risk of Bias, Imprecision, Inconsistency, Indirectness, and Publication Bias. Publication bias in this context refers to the stated values of HbA1c in the reviewed papers and the conclusions drawn from the results, since unpublished and unreferenced studies are not available. All study conclusions were carefully reviewed along with the **Supplemented Data**. To minimize the risk of including predominantly “positive” analyses, we aimed to include also government-sanctioned assessments, which were available online, such as IQWiG reports, and reports by the US AHRQ (Agency for Health Research and Quality).

## Results

### Search Results

After the search in PubMed, and EMBASE with key words diabetes and new technologies 1,495 studies were screened based on abstracts. Then we performed the second search in PubMed and Cochrane library with the terms diabetes, systematic review, and new technologies and we identified 300 studies. We excluded 195 due to duplication and 1,216 due to non-correspondence with the inclusion criteria, and 84 studies were reviewed manually. After adding the term meta-analysis, we expand the list to 138 studies. Out of them 56 candidate full texts were reviewed a third time by an independent reviewer, and a further 24 were excluded. There are multiple reasons for excluding studies, which are all summarized in **Supplementary Table 1**, some of the reasons are systematic review of the application of the technologies, not purely on effectiveness, systematic reviews commissioned by regulatory bodies, which did not provide all evidence, but just a full working summary and others. Finally, we included 32 full systematic reviews and/or meta-analyses for which the research quеstion was clearly defined (12–38, 40–44). The included studies were of high quality, predominantly focusing on randomized controlled trials. Seven of the included systematic reviews included also observational studies assessing the effect of DMS in everyday clinical practice.

**Figure 2:**
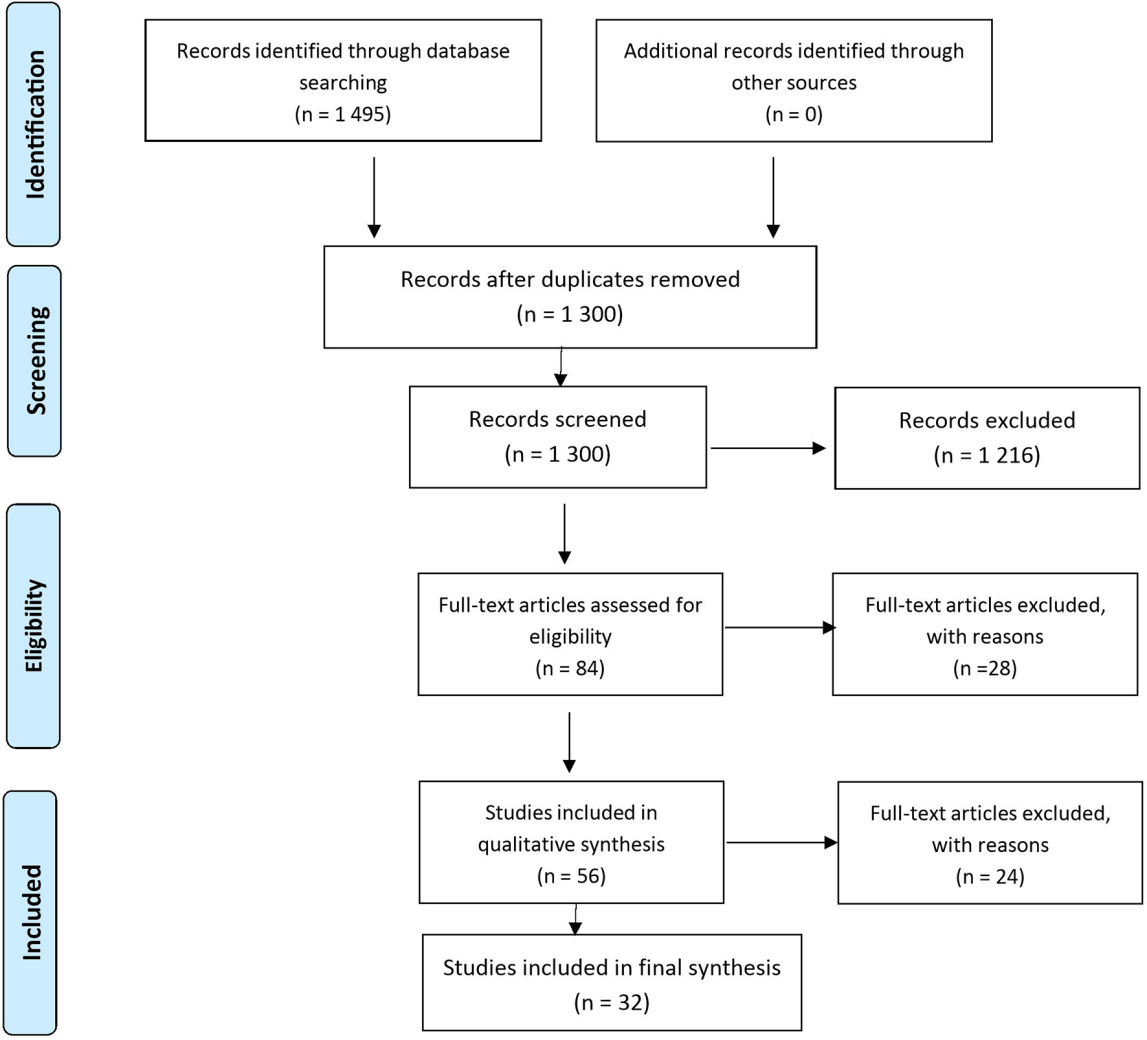
PRISMA 2009 Flow Diagram.

They are summarized in **Table 1**. A narrative synthesis is presented. **Figure 2** presents the search process flow chart.

### Characteristics of the Studies and Patient Populations

The details of the included SRs (n = 16) and SRs plus MAs (n = 16) are presented in **Table 2**. They were published in the period 2008–2019 and the number of analyzed studies (randomized clinical trials, cohort, crossover or parallel or prospective observational studies, etc.) in each separate SR or MA varied between 2 and 44. Diabetes monitoring systems observed were: Continuous Glucose Monitoring (CGMS), Sensor Augmented Pump Therapy (SAPT), Self-monitoring Blood Glucose (SMBG), Continuous subcutaneous insulin infusion (CSII), Flash Glucose Monitoring (FGM) or Intermittent-scanned continuous glucose monitoring (isCGM), Closed-loop systems, and telemedicine. All studies analyzed and reported the changes in glycated hemoglobin (HbA1c) levels as a primary clinical outcome. Some of them reported results about well-being, hypoglycemic fear and episodes, hyperglycemia incidents, overall diabetes distress, quality of life, patients’ satisfaction. Standard care (for example a weekly venipuncture protocol), conventional treatment (non-meter through urine tests and blood-glucose levels measured at the fortnightly clinic visits), or no comparator was used mainly as comparators for all CGMS. CGMSs were also compared with SMBG with or without CSII.

**Figure 3:**
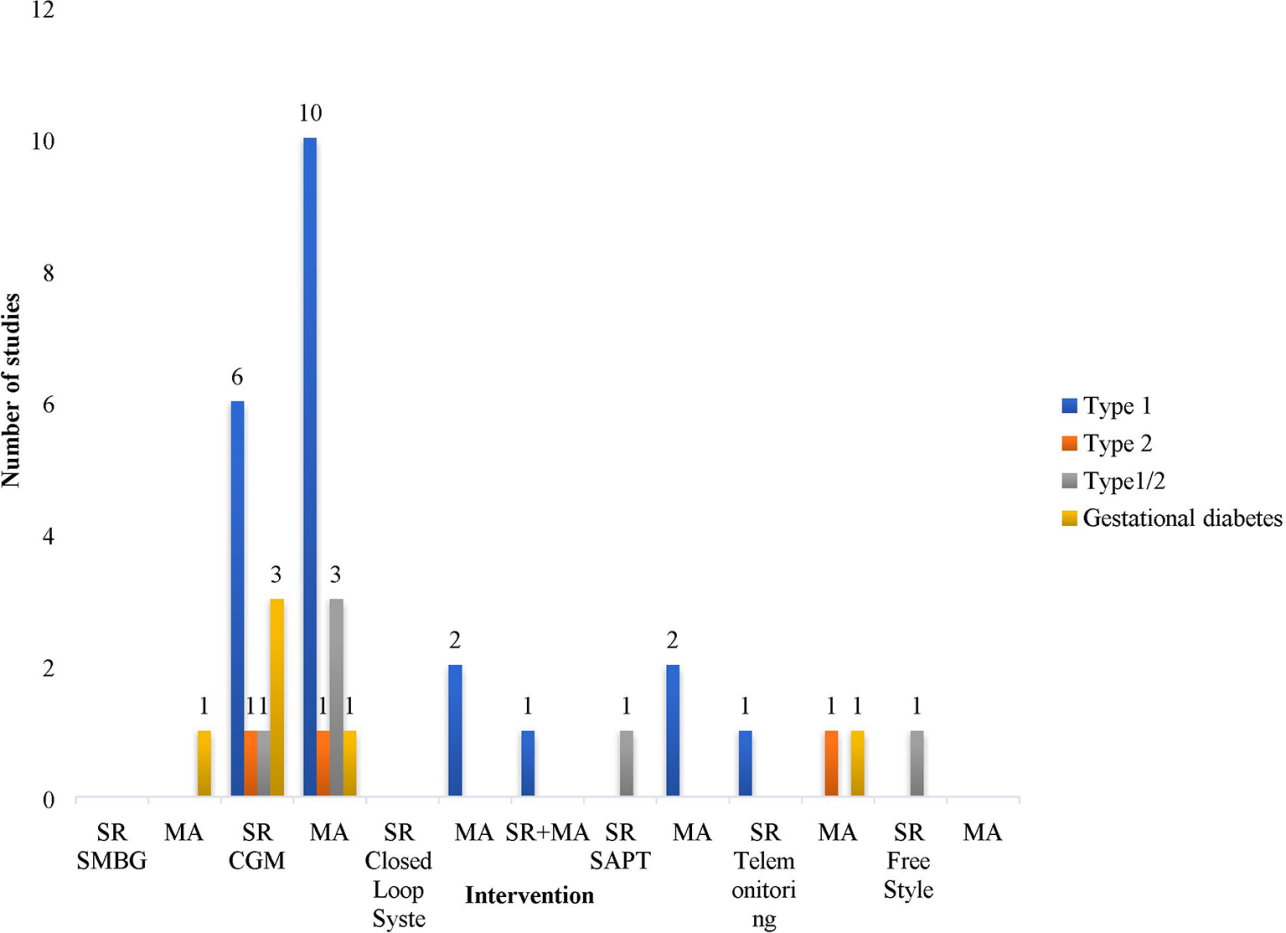
Number of studies presented by type of intervention and type of diabetes.

**Figure 4:**
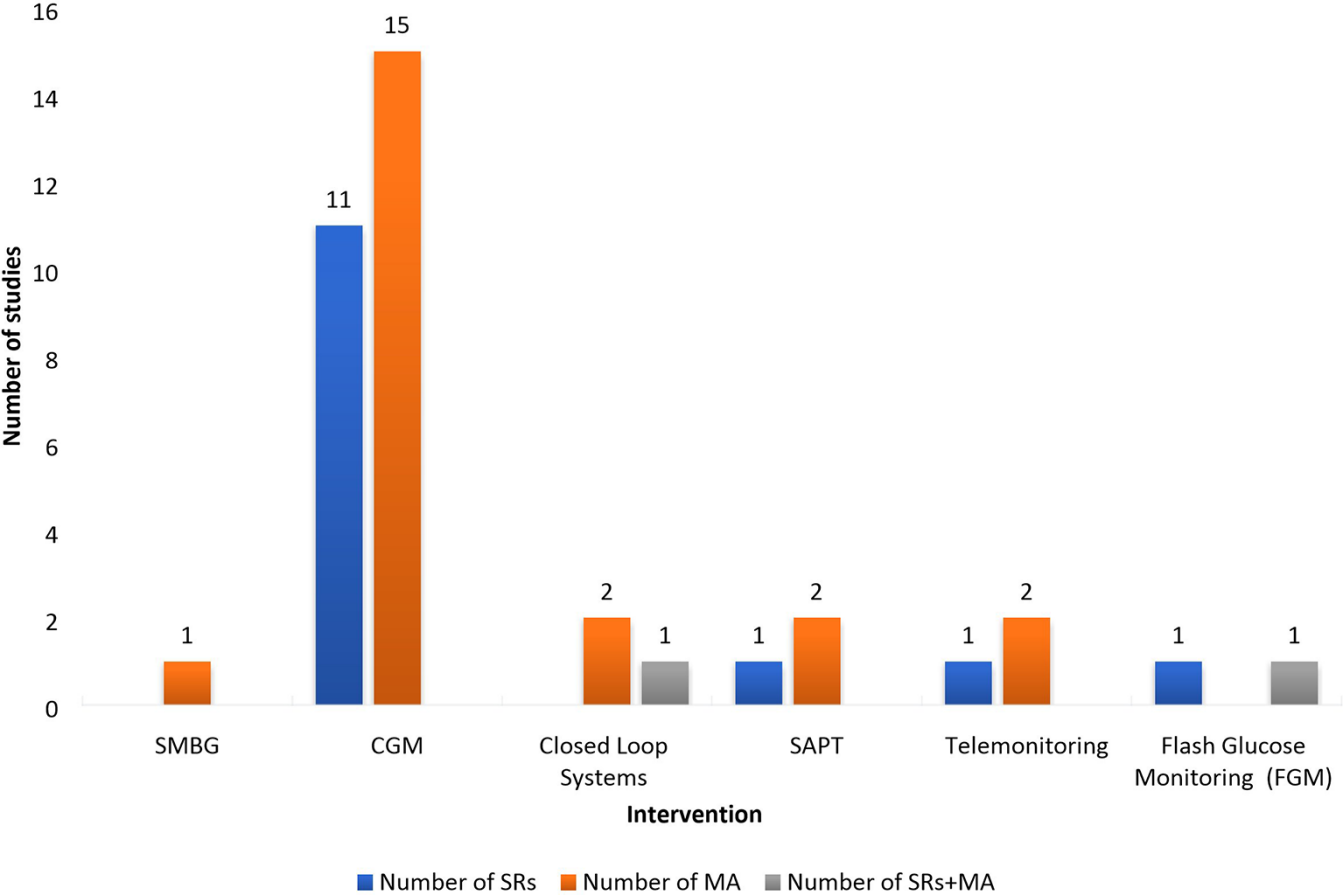
Number of studies presented by type of intervention and type of studies. SMBG, Self-monitoring Blood Glucose; CGM, Continuous Glucose Monitoring; MA, Meta-Analysis; SAPT, Sensor Augmented Pump Therapy; SR, Sytematic review.

The technologies, that were assessed in the analyzed systematic reviews and meta-analyses, and the relevant number of studies identified are presented on **Figures 3** and **4**. The most reviewed technologies were the continuous glucose monitoring systems with 11 systematic reviews and 15 meta-analyses. One analysis focused on self-monitoring approaches. It should be noted that CGMS studies also evaluated continuous subcutaneous injections as a method of insulin delivery and had many analyzed subsections. Most analyses focused on Type 1 diabetes (26) with only a small amount analyzing separately intensive insulin control in Type 2 patients, or in a mixed sample of Type 1 and Type 2 diabetes patients. The number of studies investigating gestational diabetes patients are limited (6) as they mostly compare SMBG *vs.* CGM and SMBG *vs.* standard care.

### Clinical Outcomes

Blood glucose levels are an important clinical measure for estimating effectiveness, and overall analysis of all included studies showed that there is a reduction in HbA1c levels. These can be seen in both Type 1 and Type 2 diabetes, for all interventions.The reductions are indeed almost negligble. Since the aim of control, even self-control of diabetes mellitus (DM), is to reduce HbA1c levels, the purpose of the new diabetes technologies, apart from blood glucose control is to reduce glycemic excursions, hypo- or hyperglycemia episodes, increase time spent within desired ranges, and improve compliance, especially in children. The reduction in glycated hemoglobin (HbA1c) levels varies from 0·17 to 0·70% during use of CGM. It is most significant for CGM identified in meta-analysis—from 0·23 to 0·37% for patients with Type 1 diabetes and from 0·20 to 0·48% for Type 2 diabetes. In systematic reviews a wider range and greater reduction of HbA1c were observed: from 0·20 to 0·70% in Type 1 and from 0·17 to 0·50% in Type 2. Telemonitoring would support the reduction of the HbA1c-levels in patients with Type 1 diabetes from 0·53 to 1·10%. Data for gestational diabetes was identified only for CGM where the reduction is 0·26–0·34% (23). Change in QALY (ΔQALY) was observed only in one study (19) and it is higher in Type 2 diabetes (0·272) than in Type 1 diabetes (0·046) when comparing real-time CGM *vs.* SMBG.

When using CGM the time spent in hypoglycemia is expected to be lower *vs.* SMBG—0·4–0·1 h/day *vs.* 0·65–0·6 h/day (31). Hill et al. (20), reported significant reduction in time spent in hyperglycemia—the mean difference was −68.56 min/day favoring RT-CGM (95% CI, −101·17 to −35·96) *vs.* SMBG. CGM ensured the rate for hypoglycemia of 3·50 (95% CI, 1·07 to 11·44) and for hyperglycemia 1·42 (95% CI, 0·26 to 7·82) when comparing SMBG and CGM. Significant decrease in time in hypo- and hyperglycemia for closed-loop systems in comparison with CSII was observed—mean difference of 0·67 and 3·01%, respectively (16).

## Discussion

### Summary of Results

We took a much broader approach of including systematic reviews of randomized clinical trials (RCTs) and observational studies because regulatory authorities prefer to have a real-world evidence for the decision making when they decide to reimburse a particular device. The most notable conclusions, with the level of evidence used are summarized in **Table 2**. Since we predominantly focused on SRs and MAs of randomized trials, the strength of evidence for most studies is high, such as in DMS, where 16 of the 26 studies were of high quality and 9 of moderate. We used the GRADE system to assess study quality, and it should be noted, that GRADE includes some subjectivity, since it is implemented manually and not mechanically. Although the GRADE system is transparent, the decision ultimately falls on the reviewer whether to downgrade a randomized trial based on the GRADE system’s “factors which may influence the quality level of a body of evidence.” The closed-loop systems significantly reduce complications such as hypo- or hyperglycemia, as well as the CGMs, which although moderately effective, increased the time spent in range regardless if the monitoring system was with self-injection or continuous injection delivery methods. Patients with high-compliance benefit less than patients with low compliance. Based on the collected evidence the current systematic review could highlight that the CGM is an effective and suitable method for monitoring of blood glucose levels. It could ensure reductions in HbA1c as they vary in a wide range—between 0.20–0.70% in Type 1 and between 0.17 and 0.50% in Type 2 diabetes patients. The accuracy and benefits of CGM utilization are deeply examined and confirmed. The evidences reveal that real-time CGM in Type 1 diabetes improves clinical parameters, whereas a smaller number of studies consider the results of patients with Type 2 diabetes. The recommendations mainly concern improvement of unforeseen hypoglycemia risk and glucose variability in Type 2 diabetes patients (49, 51). In our study we also found that larger number of analysis are focused on Type 1 diabetes, while those analyzing the results in Type 2 or combined studies are a smaller number.

The latest years precision of CGM systems has improved and their accuracy within glucose levels >80–200 mg/dl is similar (46). CGM could be used for self-adjustment of dosage, interpretation of hypoglycemia results, and measurement of response to therapy as it covers a wide range of glucose values. Its accuracy depends also on glucose levels variability (39). CGM is likely to improve treatment results, improve glycemic control and quality of life, as well as lower micro- and macro-vascular outcomes despite the existing barriers and educational needs for physicians and patients (55, 65, 66). The utilization of CGM with remote monitoring in children with Type 1 diabetes leads to better quality of life, parental sleep, and decreases family stress (56), while utilization in youth with Type 1 diabetes resulted in improved adherence, glycemic control, as well as a low psychosocial distress (57). Moreover, CGM is able to pick up asymptomatic hypoglycemic episodes in older patients with diabetes Type 1 or 2 and to ensure a reduction in severe hypoglycemic episodes (47). Argento et al. reported that the severe hypoglycemic episodes dropped from 52 (5 years before CGM initiation) to 12 after starting CGM (50). The proportion of patients with Type 1 diabetes with any severe hypoglycemia felt from 79 to 31% after initiation of CGM. However, Lagarde et al. concluded that no difference exists between the number of minor hypoglycemic episodes between the CGMS and the control group of children with Type 1 diabetes (mean difference 0·53, 95% CI, −0·68 to 1·74; p = 0·39) (47). Similar results are presented by Langendam et al. —no significant difference is revealed in risk of severe hypoglycemia or ketoacidosis between CGM and SMBG adults with Type 1 diabetes (29). However, due to the small number of participants and limitations, findings should be interpreted with significant caution. Hill et al. did not find any significant difference in severe hypoglycemia events between the rt-CGM and SMBG groups, but found a significant reduction in time spent in the hyperglycemic range (31). Decreased time in hypoglycemia (13 out of 15 studies) in Type 1 diabetes as well as increased time in range (TIR) as a result of CGM usage were also observed in a systematic review by De Ridder et al. (33). Other study reported that adult patients with Type 1 diabetes who use CGM perceive improvements in their quality of life, especially related to hypoglycemia fear (22). Only a few studies have found positive outcomes regarding hypoglycemia when using CGM (reduction in nocturnal hypoglycemia episodes by 54% with is CGM *vs.* SMBG (−0·29 ± 0·08 h per 7 h; P = 0·0001) (45, 63) but no decrease in time spent in hypoglycemia was observed. The other important outcome, Time in Glycemic Range (70–180 mg/dl), is reported to increase with isCGM (intermittently scanned) among well-controlled patients with Type 1 diabetes (33). Whereas, the results are controversial among adult patients with uncontrolled T2DM using insulin (62).

Jones et al., reported that CGM is able to reduce neonatal hypoglycemia (RR 0·66, 95% CI 0·48 to 0·93; 3 studies, 428 infants) (21). Latest updated evidence from 2019 by Yu et al. suggests that CGM is superior to SMBG among pregnant women with gestational diabetes mellitus as it is able to detect hypoglycemic and hyperglycemic episodes (36). Therefore, regular monitoring of glucose levels in pregnant women with diabetes through specific glucose monitoring devices could ensure limitation of hypoglycemic episodes and then influence the outcomes for both mother and child. No significant improvement in the frequency of neonatal hypoglycemia or any other primary outcomes were detected among pregnant women with pre-existing diabetes when using glucose monitoring technique.

Our study reveals a small number of MAs and SRs exploring results in CGM utilization in children. The findings show that CSII was associated with improved quality of life compared with MDI and similar results on HbA1c levels and severe hypoglycemia. Regarding closed-loop systems, meta-analyses by Karageorgiou et al., and by Weisman et al., showed that these systems lead to significantly higher percentage of time spent in the target glycemic range and to lower percentages of time in hyperglycemia and hypoglycemia for non-adult Type 1 diabetes patients (16, 17). Free Style Libre Flash Glucose Self-Monitoring System also showed promising results for reduction in time and number of events with glucose levels <3·9 in 24 h in comparison with SMBG. Evidence regarding isCGM (Intermittent-scanned continuous glucose monitoring) impact on improving time in glycemic range, glycemic variability, and hypoglycemia are variable and further clinical trials should investigate these devices (12).

Because of the variability and lack of enough strong evidence, no general conclusion or recommendation about the patients target groups who might be most suitable for particular DMS could be highlighted. Basing on the available evidence, it could be mentioned that patients’ satisfaction, preferences, lifestyle habits, age, therapy applied, and severity of the condition (type of diabetes, duration, concomitant diseases, etc.) are some of the main criteria for choosing a method for monitoring and control.

### Limitations and Strengths

The current systematic review has significant strengths as it gathers evidence for the effectiveness of a variety of diabetes monitoring devices both from controlled interventional studies and observational studies from the everyday clinical practices thus providing the opportunity to assess the effects of DMS from the perspective of different study desings. Moreover, a wide range of diabetes patients were included in the analysis—diagnosed with Type 1, Type 2, or gestational diabetes. Multiple databases were searched to identify relevant studies which answer to the research question. This systematic review of reviews provides evidence to inform both clinical practice and future research.

The main limitation is that the number of evidences for some diabetes monitoring systems such as telemonitoring, closed-loop systems, and SAPT, is too narrow, not sufficiently enough, and lack of statistical significance to make general conclusions. Moreover, different outcomes are measured and compared in the different studies which is a strong complication for a more comprehensive synthesis and analysis. Because of the limited data for assessing the effectiveness of monitoring technologies only the reduction in HbA1c levels for some DMS was analyzed. There are other criteria for assessing the quality of glycemic control such as number, duration of hypo- and hyperglycemic episodes, and time in glycemic range. Due to insufficient and controversial evidence for all valuable parameters, we assessed and presented only the variability in HbA1c for different patients’ groups and by type of study analysed (SR or SR+MA). Moreover, due to the heterogeneity of the methodologies, patient populations, and gathered data, we were not able to perform formal meta-analysis. So, a narrative synthesis is presented which could also be highlighted as a strong limitation of the study. Some DMS are innovative and not commonly applied so there is not enough relevant evidence. No studies comparing FGM and rt-CGM were included and analyzed in the current review which could be highlighted as another limitation of the review.

We made a distinction between the studies only on the criteria of whether they are only systematic reviews or include and meta-analysis of the analysed studies. No other selection criteria were applied for the primary studies included in each one of the observed reviews. We recognize that it might be a limitation of our analysis but trusted the authors performing the systematic reviews in their proper selection of the comparable RCTs or observational studies.

### Comparison With Other Studies

Very few studies performing a systematic review of already published SR and SR+MA of continuous glucose monitoring systems were identified. Published studies focus mainly on specific group of patients with Type 1 or Type 2 diabetes and on specific intervention (58).

However, a similar approach of systematizing published MA assessing supported self-management for people with Type 2 diabetes was identified. The authors focus on the role of self-management mechanism as one of main factors affecting the treatment outcomes and quality of life of patients. Some of the studies in the systematic review of meta-analysis show that self-monitoring systems and tele-health may provide some advantages in the process of self-management. Authors’ conclusions focus mainly on informative type of these findings which could be in favor to policy makers and health care professionals (59).

Other systematic review of reviews evaluates technology-enabled diabetes self-management. The study shows that mobile technologies for self-management of diabetes improve patient-generated health data and communication between patients and health care professionals. The results show that technology-enabled diabetes self-management solutions significantly improve HbA1c (67).

Similar to our results, a conducted narrative review showed that use of CGM in Type 2 diabetes patients leads to greater reductions in HbA1c in comparison with traditional self-monitoring as higher compliance to CGM was also reported. Logically, addition of other methods to CGM such as lifestyle counseling could lead to further improvements (52).

Evidences of key publications associating CGMS reported improvement in clinical outcomes, reducing of hypoglycemia and impact on physical, emotional, and relational aspects of everyday life (48). The positive effects of CGMS utilization are discussed from American Association of Clinical Endocrinologists and the American College of Endocrinology. The conclusion they reach is that increasing utilization of CGMS will probably improve the health outcomes, decrease health care resource costs for acute and chronic complications (53).

A review on studies observing glucose management reported that according to current literature evidence utilization of CGMS is mainly recommended in T1DM patients with a poor control of HbA1c levels after SMBG and risk of hypoglycemia, which confirms our findings. Utilization of CGMS is favored for patients with Type 2 diabetes who reported severe hypoglycemia or suspected hypoglycemia, particularly nocturnal (64).

We found very few studies reporting the M-value (durable nyctohemeral measurement of glycemic behavior) (16, 17) and SD of 24-h glucose (24, 32, 33) and did not find a study reporting the Mean amplitude of glycemic excursion (MAGE). We recognize that those measures of continuous diabetes control are currently introduced and might be more informative for the endocrinologist. Further studies need to be done to explore the previous research/systematic reviews investigating the effect of DMS on indices of glycemic variability such as MAGE, M-value, and SD of average 24-h glucose concentrations in patients with diabetes.

Only a few studies, however, assess the role of meta-analyses and systematic reviews on diabetes monitoring systems and their efficacy in terms of the HTA perspective. Insufficient cost-effectiveness studies and randomized clinical trials in specific patient populations used in HTA resulted in different criteria and rate of reimbursement among countries (54). In 2018 EUnetHTA published an HTA core model for rapid relative effectiveness assessment of continuous glucose monitoring (CGM real time, rtCGM) and flash glucose monitoring (FGM) as personal, standalone system in patients with diabetes mellitus treated with insulin (60). This report shows that in the light of the increasing number of different systems for rtCGM available on the market, systematic reviews and meta-analyses assessing their relative clinical effectiveness could be of a great importance in the assessment of their cost-effectiveness in terms of decision making. These studies could provide a summary of the best scientific available evidence which could facilitate the appraisal process and decision making and could favor the national/regional/local HTA. Health Technology Wales evidence appraisal report also included systematic review on the clinical and cost-effectiveness when providing decision for FreeStyle Libre flash glucose monitoring for the management of Type 1 or Type 2 diabetes (61).

## Future Studies

Future studies should be performed to evaluate each technology for all subgroups of patients, since preliminary results showed also that effectiveness is better in adults than in children. In children, however, the habituation with the devices is better which determines better compliance. These findings could also facilitate not only the process of patient-centered care but also could provide methodologies for personalized information on the effectiveness and cost-effectiveness of such health technologies thus improving the decision making process in terms of reimbursement.

## Conclusions

Current systematic review of already published systematic reviews and meta-analyses suggests that no statistically significant difference exists between the values of HbA1c as a result of application of any type of DMS. The most notable are the changes in HbA1c for patients with Type 1 diabetes using CGM devices. The number of hypo- and hyperglycemic episodes and Time in Glycemic Range are some of the most valuable outcomes that should be considered when choosing the most appropriate diabetes monitoring system for each patient. Undoubtedly, as the American Diabetes Association currently recommends, CGM are most suitable for diabetes patients with insufficient and unsatisfied disease control and high risk of hypoglycemia. However, due to the diversity of the results about the real effectiveness of DMS, future more comprehensive studies assessing the effectiveness, cost-effectiveness, and comparative effectiveness of DMS, stratifying the patients in different subgroup, are needed.

## Data Availability Statement

The original contributions presented in the study are included in the article/**Supplementary Material**. Further inquiries can be directed to the corresponding author.

## Author Contributions

All the authors have provided valuable contributions to the manuscript. Conceptualization: GP, WG, GG-S. Formal analysis: MK, KT, MD, ZM, GP. Methodology: GP, MD, ZM. Project administration: GP, WG. Writing: MK, KT, MD, ZM, GP. Writing—review and editing: WG, GG-S, KT, MH. Validation: WG, GG-S, MH, MK. All authors contributed to the article and approved the submitted version.

## Funding

The investigation has received funding from the European Union’s Horizon 2020 research and innovation programme under grant agreement No 825162: “HTx: Next Generation Health Technology Assessment to support patient-centered, societally oriented, real-time decision-making on access and reimbursement for health technologies throughout Europe” (H2020-825162, 2019-2023).

## Conflict of Interest

The authors declare that the research was conducted in the absence of any commercial or financial relationships that could be construed as a potential conflict of interest.
