# Supplementary material for: A Systematic Review of Collective Evidences Investigating the Effect of Diabetes Monitoring Systems and Their Application in Health Care
Source: Front Endocrinol (Lausanne). 2021 Mar 16;12:636959. doi: 10.3389/fendo.2021.636959 (PMC8008960; doi:10.3389/fendo.2021.636959)
Supplement: Supplementary file 1 [file Table_1.docx]

**Supplementary table 1.** Excluded systematic reviews

| ***REVIEW*** | ***REASON FOR EXCLUSION*** |
| --- | --- |
| Yamada T et al 2018 ^[[1]](#endnote-1)^ | Not meeting the inclusion criteria. The SR evaluates the efficacy of SGLT2 antagonists as an add-on therapy to insulin in type 1 diabetes |
| Fahy BG et al 2009 ^[[2]](#endnote-2)^ | Not meeting the inclusion criteria to be a systematic review |
| Asrani NA et al 2019 ^[[3]](#endnote-3)^ | Not meeting the inclusion criteria. The SR evaluates the cutaneous complications with CGM |
| MacLeod SF et al 2013 ^[[4]](#endnote-4)^ | Not meeting the inclusion criteria. The SR evaluates the effect on exercise on the postprandial glucose |
| Price D^[[5]](#endnote-5)^ | Not meeting the inclusion criteria. The study reviews limitations of SRMA in CGM publications |
| Shalitin S. ^[[6]](#endnote-6)^ | Not meeting the inclusion criteria to be a systematic review. |
| Peyrot M^[[7]](#endnote-7)^ | Not meeting the inclusion criteria to be a systematic review. |
| Peters AL^[[8]](#endnote-8)^ | Not meeting the inclusion criteria to be a systematic review. |
| Srinivasan V^[[9]](#endnote-9)^ | Not meeting the inclusion criteria in regards to examined technologies. |
| Pease A^[[10]](#endnote-10)^ | Not meeting the inclusion criteria. The study provides information about Protocol for SR |
| Pamungkas R^[[11]](#endnote-11)^ | Not meeting the inclusion criteria in regards to examined technologies. |
| Juvenile Diabetes Research Foundation^[[12]](#endnote-12)^ | Not meeting the inclusion criteria to be a systematic review. |
| Juvenile Diabetes Research Foundation^[[13]](#endnote-13)^ | Not meeting the inclusion criteria to be a systematic review. |
| Juvenile Diabetes Research Foundation^[[14]](#endnote-14)^ | Not meeting the inclusion criteria to be a systematic review. |
| Liberatore et al. (2006)^^[[15]](#endnote-15)^^ | Not meeting the inclusion criteria to be a systematic review. The study describes the process of development, application, response and complications related to insulin pumps. |
| Golden et al. (2012) ^[[16]](#endnote-16)^ | Present and summarize the results from another study already included in the review. |
| Messer et al. (2017)^[[17]](#endnote-17)^ | It is a qualitative not quantitative meta-synthesis. |
| Al Tulaihi, et al (2017) ^[[18]](#endnote-18)^ | Considers only new medicines. |
| Armaghanian N., et al.(2016) ^[[19]](#endnote-19)^ | Patients do not have diabetes but cystic fibrosis. |
| Fullerton B, (2014)^[[20]](#endnote-20)^ | Compares only dosing regimens of insulin. |
| Bell Kirstine J., (2015) ^[[21]](#endnote-21)^ | Considers only dietetic regimens. |
| Shalitin S., et al (2011)^[[22]](#endnote-22)^ | Review but not systematic. |
| van Steen et al.(2017) ^[[23]](#endnote-23)^ | Not diabetic patients |
| Sinclair JC et al. (2011) ^[[24]](#endnote-24)^ | No technology, only therapeutic interventions |
|  |  |

1. Yamada T, Shojima N, Noma H, Yamauchi T, Kadowaki T. Sodium-glucose co-transporter-2 inhibitors as add-on therapy to insulin for type 1 diabetes mellitus: Systematic review and meta-analysis of randomised controlled trials. Wiley 2018; DOI:10.11111/dom.13260 [↑](#endnote-ref-1)
2. Fahy BG, Sheehy AM, Coursin DB. Glucose control in the intensive care unit. Crit Care Med 2009; 37(5):1769-1776 [↑](#endnote-ref-2)
3. Asarani AM, Reynolds AN, Boucher SE, de Bock M, Wheeler BJ. Cutaneous complications with continuous or flash glucose monitoring use: systematic review of trials and observational studies. Journal of diabetes science and technology 2019. DOI: 10.1177/1932296819870849 [↑](#endnote-ref-3)
4. MacLeod SF, Terada T. Exercise lowers postprandial glucose but not fasting glucose in type 2 diabetes: a meta-analysis of studies using continuous glucose monitoring. Diabetes Metab Res Rev 2013; 29:593-603. DOI: 10.1002/dmrr.2461 [↑](#endnote-ref-4)
5. Price D, Graham C, [Parkin CG](https://www.ncbi.nlm.nih.gov/pubmed/?term=Parkin%20CG%5BAuthor%5D&cauthor=true&cauthor_uid=26420626), [Peyser TA](https://www.ncbi.nlm.nih.gov/pubmed/?term=Peyser%20TA%5BAuthor%5D&cauthor=true&cauthor_uid=26420626).Are Systematic Reviews and Meta-Analyses Appropriate Tools for Assessing Evolving Medical Device Technologies? [J Diabetes Sci Technol.](https://www.ncbi.nlm.nih.gov/pubmed/26420626) 2015 Sep 29;10(2):439-46. doi: 10.1177/1932296815607863. [↑](#endnote-ref-5)
6. S. Shalitin, M. Gil, R. Nimri, L. de Vries, M. Y. Gavan, M. Phillip Predictors of glycaemic control in patients with type 1 diabetes commencing continuoussubcutaneous insulin infusion therapy Diabet Med 2010; 27: 339–47 [↑](#endnote-ref-6)
7. [Peyrot M](https://www.ncbi.nlm.nih.gov/pubmed/?term=Peyrot%20M%5BAuthor%5D&cauthor=true&cauthor_uid=19132857), [Rubin RR](https://www.ncbi.nlm.nih.gov/pubmed/?term=Rubin%20RR%5BAuthor%5D&cauthor=true&cauthor_uid=19132857). Patient-reported outcomes for an integrated real-time continuous glucose monitoring/insulin pump system. [Diabetes Technol Ther.](https://www.ncbi.nlm.nih.gov/pubmed/19132857) 2009 Jan;11(1):57-62. doi: 10.1089/dia.2008.0002. [↑](#endnote-ref-7)
8. [Peters AL](https://www.ncbi.nlm.nih.gov/pubmed/?term=Peters%20AL%5BAuthor%5D&cauthor=true&cauthor_uid=27588440), [Ahmann AJ](https://www.ncbi.nlm.nih.gov/pubmed/?term=Ahmann%20AJ%5BAuthor%5D&cauthor=true&cauthor_uid=27588440), [Battelino T](https://www.ncbi.nlm.nih.gov/pubmed/?term=Battelino%20T%5BAuthor%5D&cauthor=true&cauthor_uid=27588440), [Evert A](https://www.ncbi.nlm.nih.gov/pubmed/?term=Evert%20A%5BAuthor%5D&cauthor=true&cauthor_uid=27588440), [Hirsch IB](https://www.ncbi.nlm.nih.gov/pubmed/?term=Hirsch%20IB%5BAuthor%5D&cauthor=true&cauthor_uid=27588440), [Murad MH](https://www.ncbi.nlm.nih.gov/pubmed/?term=Murad%20MH%5BAuthor%5D&cauthor=true&cauthor_uid=27588440), [Winter WE](https://www.ncbi.nlm.nih.gov/pubmed/?term=Winter%20WE%5BAuthor%5D&cauthor=true&cauthor_uid=27588440), [Wolpert H](https://www.ncbi.nlm.nih.gov/pubmed/?term=Wolpert%20H%5BAuthor%5D&cauthor=true&cauthor_uid=27588440).Diabetes Technology-Continuous Subcutaneous Insulin Infusion Therapy and Continuous Glucose Monitoring in Adults: An Endocrine Society Clinical Practice Guideline. [J Clin Endocrinol Metab.](https://www.ncbi.nlm.nih.gov/pubmed/27588440) 2016 Nov;101(11):3922-3937. Epub 2016 Sep 2. [↑](#endnote-ref-8)
9. [Srinivasan V](https://www.ncbi.nlm.nih.gov/pubmed/?term=Srinivasan%20V%5BAuthor%5D&cauthor=true&cauthor_uid=24783254), [Agus MS](https://www.ncbi.nlm.nih.gov/pubmed/?term=Agus%20MS%5BAuthor%5D&cauthor=true&cauthor_uid=24783254).Tight glucose control in critically ill children--a systematic review and meta-analysis.[Pediatr Diabetes.](https://www.ncbi.nlm.nih.gov/pubmed/24783254) 2014 Mar;15(2):75-83. [↑](#endnote-ref-9)
10. [Pease A](https://www.ncbi.nlm.nih.gov/pubmed/?term=Pease%20A%5BAuthor%5D&cauthor=true&cauthor_uid=29530081), [Lo C](https://www.ncbi.nlm.nih.gov/pubmed/?term=Lo%20C%5BAuthor%5D&cauthor=true&cauthor_uid=29530081), [Earnest A](https://www.ncbi.nlm.nih.gov/pubmed/?term=Earnest%20A%5BAuthor%5D&cauthor=true&cauthor_uid=29530081), [Liew D](https://www.ncbi.nlm.nih.gov/pubmed/?term=Liew%20D%5BAuthor%5D&cauthor=true&cauthor_uid=29530081), [Zoungas S](https://www.ncbi.nlm.nih.gov/pubmed/?term=Zoungas%20S%5BAuthor%5D&cauthor=true&cauthor_uid=29530081).Evaluating optimal utilisation of technology in type 1 diabetes mellitus from a clinical and health economic perspective: protocol for a systematic review.[Syst Rev.](https://www.ncbi.nlm.nih.gov/pubmed/29530081) 2018 Mar 12;7(1):44. doi: 10.1186/s13643-018-0706-9. [↑](#endnote-ref-10)
11. [Pamungkas](https://www.ncbi.nlm.nih.gov/pubmed/?term=Pamungkas%20RA%5BAuthor%5D&cauthor=true&cauthor_uid=28914815) R, [Chamroonsawasdi](https://www.ncbi.nlm.nih.gov/pubmed/?term=Chamroonsawasdi%20K%5BAuthor%5D&cauthor=true&cauthor_uid=28914815) K,  [Vatanasomboon](https://www.ncbi.nlm.nih.gov/pubmed/?term=Vatanasomboon%20P%5BAuthor%5D&cauthor=true&cauthor_uid=28914815) PA Systematic Review: Family Support Integrated with Diabetes Self-Management among Uncontrolled Type II Diabetes Mellitus Patients[BehavSci (Basel)](https://www.ncbi.nlm.nih.gov/pmc/articles/PMC5618070/). 2017 Sep; 7(3): 62. [↑](#endnote-ref-11)
12. Juvenile Diabetes Research Foundation. Prolonged nocturnal hypoglycaemia is common during 12 months of continuous glucose monitoring in children and adults with type 1 diabetes Juvenile Diabetes Research FoundationContinuous Glucose Monitoring Study GroupDiabetes Care 2010; 33: 1004–8 [↑](#endnote-ref-12)
13. Juvenile Diabetes Research Foundation.Continuous Glucose Monitoring Study Group.Effectiveness of continuousglucose monitoring in a clinicalcare environment. Evidencefrom the Juvenile DiabetesResearch FoundationContinuous Glucose MonitoringTrial Juvenile Diabetes Research Foundation Continuous Glucose Monitoring Study Group Diabetes Care 2010; 33: 17–22 [↑](#endnote-ref-13)
14. Juvenile Diabetes Research Foundation. Effect of continuous glucose monitoring in well-controlled type 1 diabetes Juvenile Diabetes Research FoundationContinuous Glucose Monitoring Study Group Diabetes Care 2009; 32: 1378–83 [↑](#endnote-ref-14)
15. Liberatore R, Damiani D. Insulin pump therapy in type 1 diabetes mellitus. Jornal de Pediatria. 2006;82(4):249-54: [↑](#endnote-ref-15)
16. Golden SH, Sapir T. Methods for Insulin Delivery and Glucose Monitoring in Diabetes: Summary of a Comparative Effectiveness Review. J Manag Care Pharm. 2012 Aug;18(6 Suppl):S1-17. [↑](#endnote-ref-16)
17. Messer LH, Johnson R, Driscoll KA, Jones J.Best friend or spy: a qualitative meta-synthesis on the impact of continuous glucose monitoring on life with Type 1 diabetes. Diabet Med. 2018 Apr;35(4):409-418. doi: 10.1111/dme.13568. Epub 2017 Dec 27. [↑](#endnote-ref-17)
18. Al Tulaihi Bader, Samia Alhabib. Uncertainties around incretin-based therapies: A literature review. Saudi Pharmaceutical Journal (2017) 25, 1–7 [↑](#endnote-ref-18)
19. Armaghanian N., J.C. Brand-Miller, T.P. Markovic, K.S. Steinbeck. Hypoglycaemia in cystic fibrosis in the absence of diabetes: A systematic review. Journal of Cystic Fibrosis 15 (2016) 274–284 [↑](#endnote-ref-19)
20. Fullerton B, Jeitler K, Seitz M, Horvath K, Berghold A, Siebenhofer A. Intensive glucose control versus conventional glucose control for type 1 diabetes mellitus. Cochrane Database of Systematic Reviews 2014, Issue 2. Art. No.: CD009122. DOI: 10.1002/14651858.CD009122.pub2. [↑](#endnote-ref-20)
21. Bell Kirstine J., Carmel E. Smart, Garry M. Steil, Jennie C. Brand-Miller,Bruce King, and Howard A. Wolpert. Impact of Fat, Protein, and Glycemic Index on Postprandial Glucose Control in Type 1Diabetes: Implications for Intensive Diabetes Management in the Continuous Glucose Monitoring Era. Diabetes Care 2015;38:1008–1015 | DOI: 10.2337/dc15-0100 [↑](#endnote-ref-21)
22. Shalitin S., H. Peter Chase. Diabetes technology and treatments in the paediatric age group. Int J Clin Pract, February 2011, 65 (Suppl. 170), 76–82 [↑](#endnote-ref-22)
23. van Steen Sigrid C. J., Saskia Rijkenberg, Jacqueline Limpens, Peter H. J. van der Voort, Jeroen Hermanides and J. Hans DeVries. The Clinical Beneﬁts and Accuracy of Continuous Glucose Monitoring Systems in Critically Ill Patients—A Systematic Scoping Review. Sensors 2017, 17, 146; doi:10.3390/s17010146 [↑](#endnote-ref-23)
24. Sinclair JC, Bottino M, Cowett RM. Interventions for prevention of neonatal hyperglycemia in very low birth weight infants. Cochrane Database of Systematic Reviews 2011, Issue 10. Art. No.: CD007615. DOI: 10.1002/14651858.CD007615.pub3. [↑](#endnote-ref-24)
